# Treatment, characteristics and prognosis of central venous occlusion in hemodialysis patients with tunneled cuffed catheter dysfunction

**DOI:** 10.1080/0886022X.2026.2732516

**Published:** 2026-09-27

**Authors:** Bohua Zhang, Lichao Zhong, Yuqin Xiong, Ruoxi Liao, Tianlei Cui

**Affiliations:** Department of nephrology, West China Hospital of Sichuan University, Chengdu, Sichuan, China

**Keywords:** Hemodialysis, central venous occlusion, tunneled cuffed catheter, malfunction, prognosis

## Abstract

**Background:**

Central venous occlusion (CVO) is a severe complication in hemodialysis patients with tunneled cuffed catheters (TCC), leading to catheter dysfunction and poor outcomes. Optimal treatment remains controversial.

**Methods:**

This retrospective study included 123 hemodialysis patients with TCC dysfunction and CVO between February 2017 and December 2025. Patients underwent either exchange of the existing catheter or placement of a new catheter at a different venous site, with or without concurrent balloon angioplasty or stenting.

**Results:**

The cohort had a mean age of 61.02 ± 13.66 years, 29.2% male, with mean hemodialysis vintage of 7.86 years. Brachiocephalic vein occlusion was the most common lesion (74.8%), followed by superior vena cava occlusion (63.4%). Median catheter survival was 735 days. Recurrent dysfunction requiring reintervention occurred in 39.0% of patients (median time 255 days). Atrial thrombus (17.8%) and fibrin sheath (38.2%) were significantly associated with shorter catheter survival (*p* < 0.05). Most patients had reduced right ventricular basal diameter (98.0%) and increased left ventricular mass index (40.9%).

**Conclusions:**

Catheter exchange and endovascular treatment for central venous occlusion are safe and effective. Percutaneous superior vena cava puncture provides a valuable salvage option for this complex population, supporting durable long-term vascular access. Thrombosis and fibrin sheath predict poorer catheter survival.

## LAY SUMMARY

For patients on long-term hemodialysis, a central venous catheter (a tube placed in a large vein) is sometimes necessary when other types of vascular access are not available. However, these catheters can cause a serious complication called central venous occlusion (CVO)—a blockage in the large veins of the chest that prevents blood from flowing properly. This can lead to poor dialysis, swelling, low blood pressure, and may eventually make it impossible to continue hemodialysis treatment.

In this study, we reviewed 123 patients with CVO who underwent catheter exchange or replacement, often combined with balloon angioplasty to open the blocked vein. We found that the average time a catheter remained functional after treatment was 735 days (about 2 years). About 39% of patients needed additional intervention after a median of 255 days. The presence of blood clots in the heart or around the catheter tip was linked to shorter catheter survival.

Our study also showed that most patients had a smaller-than-normal right ventricle, while the left ventricle was often enlarged—a unique pattern that suggests the heart is affected by the vein blockage itself, rather than by fluid overload. This indicates that restoring blood flow through the blocked vein is more important than simply removing fluid by dialysis.

Overall, our findings suggest that catheter exchange with endovascular treatment is a safe and effective approach. For patients with no other options, puncturing the superior vena cava directly can serve as a valuable backup method. Recognizing and treating blood clots and fibrin sheaths early may help prolong catheter life and improve patient outcomes.

## KEY MESSAGES (IMPACT STATEMENT)

**What is known:**

Central venous occlusion (CVO) is a severe complication in hemodialysis patients with tunneled cuffed catheters (TCC), leading to catheter dysfunction and poor outcomes.

**What this study adds:**

In a cohort of 123 patients, catheter exchange with endovascular treatment achieved a median catheter survival of 735 days, with atrial thrombus and fibrin sheath identified as significant predictors of shorter survival.

**Potential impact:**

This study supports catheter exchange with endovascular intervention as a safe and effective strategy, and highlights the direct superior vena cava puncture as a valuable salvage option when conventional access is exhausted.

## Graphical abstract

**Figure:**
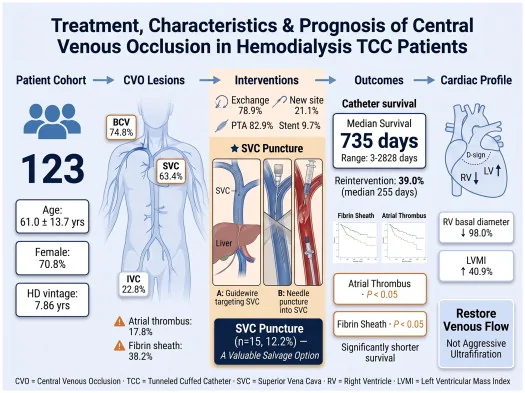

## Introduction

In patients on long-term hemodialysis, maintaining reliable vascular access is essential. Tunneled cuffed catheters (TCC) are often used when arteriovenous fistulas or grafts fail or are not possible [1,2]. However, these catheters can cause complications over time. One serious complication is central venous occlusion (CVO) [3]. Central venous occlusion blocks blood flow in large veins such as the superior vena cava, inferior vena cava, or brachiocephalic vein. This condition can lead to catheter dysfunction, poor dialysis efficiency, and patient discomfort. Symptoms of CVO may include edema and venous distension. Hypotension may also occur. However, it is not uniformly attributable to central venous occlusion and is often multifactorial. In many cases, it is incidental and related to other factors such as autonomic dysfunction, excessive ultrafiltration, or cardiac impairment. If not treated correctly, central venous occlusion can limit future access options and increase patient risk [4–6]. Ultimately, progressive CVO may lead to exhaustion of vascular access sites, resulting in difficulty maintaining or even performing hemodialysis altogether.

Several treatments are available. Endovascular therapy, particularly percutaneous transluminal angioplasty (PTA), is the recommended first-line treatment [7]. Technical success rates for endovascular treatment are excellent, however 6- and 12-month primary patency rates are poor, at 50% and 25%, respectively [4–8]. Previous studies also provided other alternative options including HeRO (Hemodialysis Reliable Outflow), computed tomography-guided translumbar catheter, transhepatic catheter and sharp recanalization. However, HeRO, translumbar catheter and transhepatic catheter have a comparatively low patency rate [9–12]. And for sharp recanalization, due to the sharp nature of the surgical instruments, operation-related complications are more frequent [13]. Therefore, there is a need to find a method to place dialysis catheter with both convenience and high patency rate.

In this study, we report our experience with treating central venous occlusion in hemodialysis patients with tunneled cuffed catheter dysfunction. We focus on treatment methods, patient characteristics, and long-term prognosis. We hope these findings will help guide clinical decisions and improve patient care.

## Methods

### Study design and participants

Our study was a retrospective study. We included long-term hemodialysis patients admitted to our hospital for tunneled cuffed catheter dysfunction between February 2017 and December 2025. All patients were diagnosed with central venous occlusion. Inclusion criteria included: (1) age ≥18 years; (2) maintenance hemodialysis *via* a tunneled cuffed catheter; (3) catheter dysfunction defined as blood flow <300 mL/min; (4) occlusion of the superior vena cava, inferior vena cava, or brachiocephalic vein confirmed by digital subtraction angiography (DSA); (5) treatment with catheter exchange or new catheter insertion during the admission. Exclusion criteria: presence of tumor that could cause inferior vena cava tumor thrombus. The study followed the principles of the Declaration of Helsinki. The Biomedical Research Ethics Committee of West China Hospital of Sichuan University approved the study (approval number: 2022 Review (1037), date of approval: September 28, 2022). Due to the retrospective design, the requirement for written informed consent was waived by the ethics committee. Patient data were anonymized prior to analysis.

### Data collection

We collected data from the hospital electronic medical record system. These data included patient demographics, medical history, symptoms, medication use, preoperative echocardiography findings, vital signs, procedure details, and outcomes.

Blood pressure measurements were obtained using a standardized protocol. Patients rested in a seated position for at least 5 minutes before measurement, and three consecutive readings were taken, with the mean value recorded for analysis. Pre-procedure blood pressure was measured within 24 hours before the endovascular intervention, and post-procedure measurements were obtained between 24 and 48 hours after the procedure.

### Endovascular procedure

A single interventional physician performed all the procedures. Patients underwent either exchange of the existing tunneled cuffed catheter or placement of a new catheter at a different site.

Conventional internal jugular vein access was prioritized for catheter exchange or *de novo* tunneled cuffed catheter placement. For patients with complete central venous occlusion precluding standard internal jugular vein puncture, percutaneous superior vena cava (SVC) direct puncture was adopted as an alternative salvage access route. The selection criteria for SVC puncture included: (1) failure of conventional internal jugular vein access; (2) complete central venous occlusion preventing guidewire passage through standard routes. The procedure involved the following steps: first, we placed a guidewire in the distal SVC *via* the femoral, iliac, or hepatic vein to serve as a fluoroscopic target; second, under combined vertical and horizontal fluoroscopic guidance, we inserted a percutaneous cholecystostomy needle into the SVC; third, after confirming needle position by blood aspiration and contrast injection, we advanced a guidewire through the needle, exchanged it for a sheath, and confirmed sheath placement with contrast venography (Figure 1). We performed standard venography *via* the internal jugular vein and/or common femoral vein to assess the central veins. Next, we crossed the venous occlusion with a guidewire. We then placed a sheath under fluoroscopy. For patients with angiographically confirmed thrombosis, we administered unfractionated heparin during the procedure as a precautionary measure to reduce the risk of distal embolization during guidewire manipulation. We acknowledge that this practice is based on clinical experience rather than specific guideline recommendations, as current KDOQI guidelines do not provide explicit guidance on prophylactic systemic anticoagulation in this setting.

**Figure 1.**
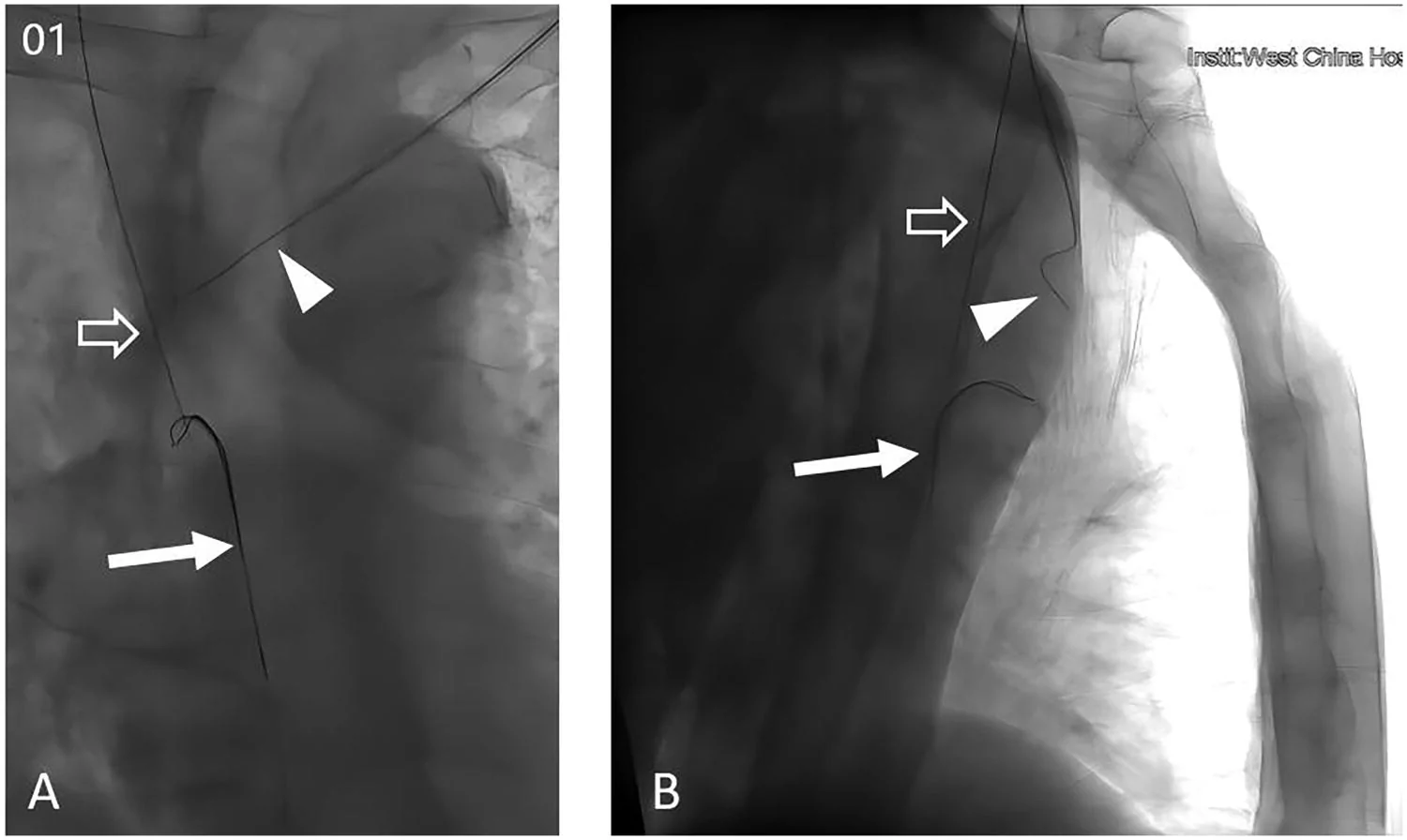
Superior vena cava puncture. The puncture needle (hollow arrow) was inserted into the superior vena cava with the guidance of two guidewires. One guidewire was inserted through the liver vein and arrived at the distal end of the superior vena cava (solid arrow), and the other guidewire was inserted through the left brachiocephalic vein and arrived at the junction of the left brachiocephalic vein and superior vena cava (triangle). The puncture needle was placed under the guidance of vertical (A) and horizontal (B) fluoroscopy.

In some patients, we performed balloon angioplasty. Balloon sizes started relatively small (e.g., 6–8 mm). We gradually increased the size to match the estimated original vein diameter (e.g., 10–15 mm). We dilated the stenotic segment for 1 to 5 min (Figures 2). For the placement of the stent, in our center, we follow a selective stenting strategy: stents are reserved only for cases with significant elastic recoil (>50% residual stenosis) after adequate balloon angioplasty. In the majority of patients, balloon angioplasty alone achieved satisfactory luminal patency. Therefore, stents were not required. Additionally, we consider the higher cost and potential long-term complications of stents, including in-stent restenosis and stent fracture, when making this decision (Figure 3).

**Figure 2.**
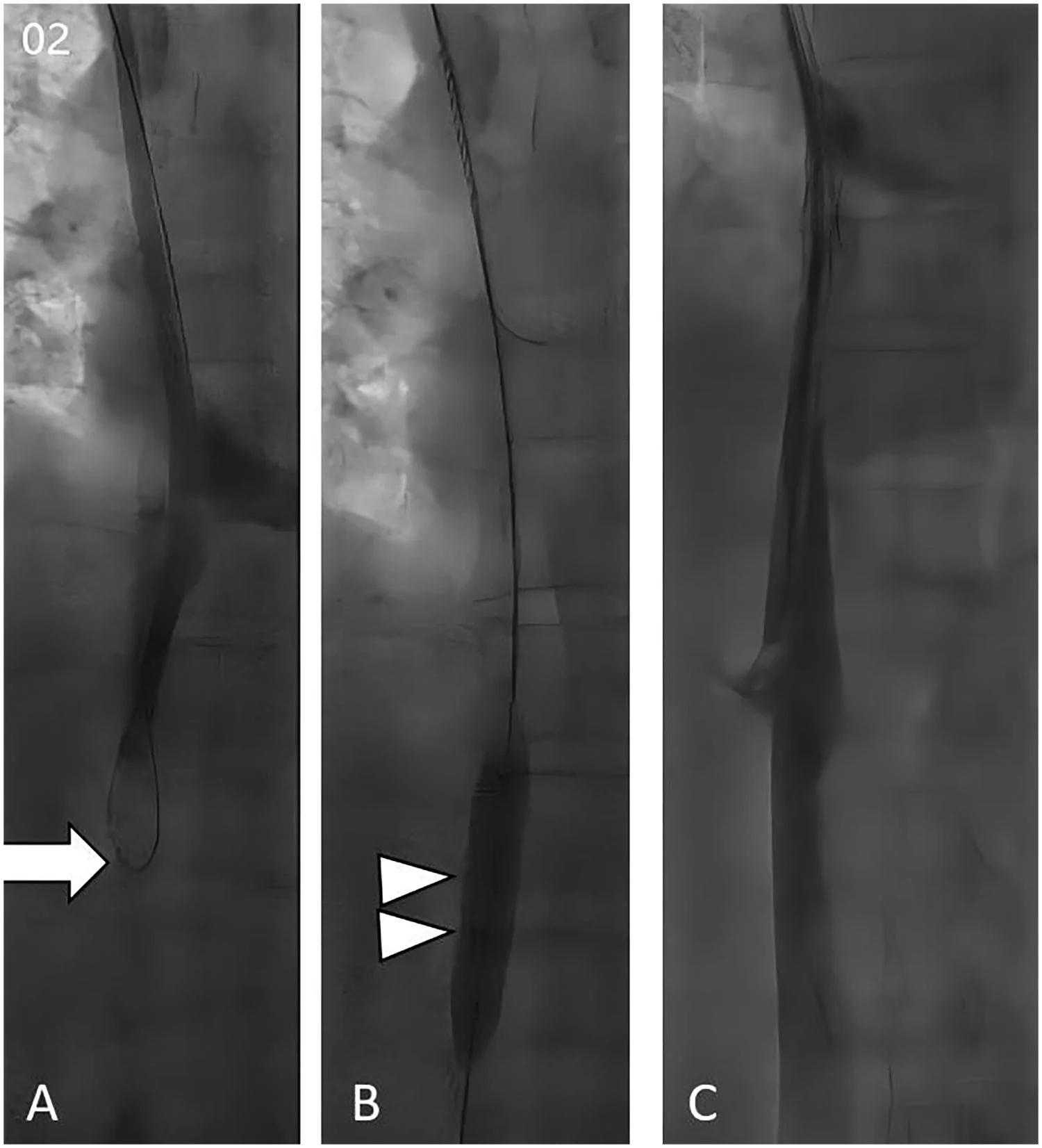
Balloon angioplasty of the inferior vena cava. A. Digital subtraction angiography showed inferior vena cava obstruction (arrow). B. Balloon angioplasty was performed at the stenosis location (triangle). C. Venography after angioplasty showed that inferior vena cava flow recovered.

**Figure 3.**
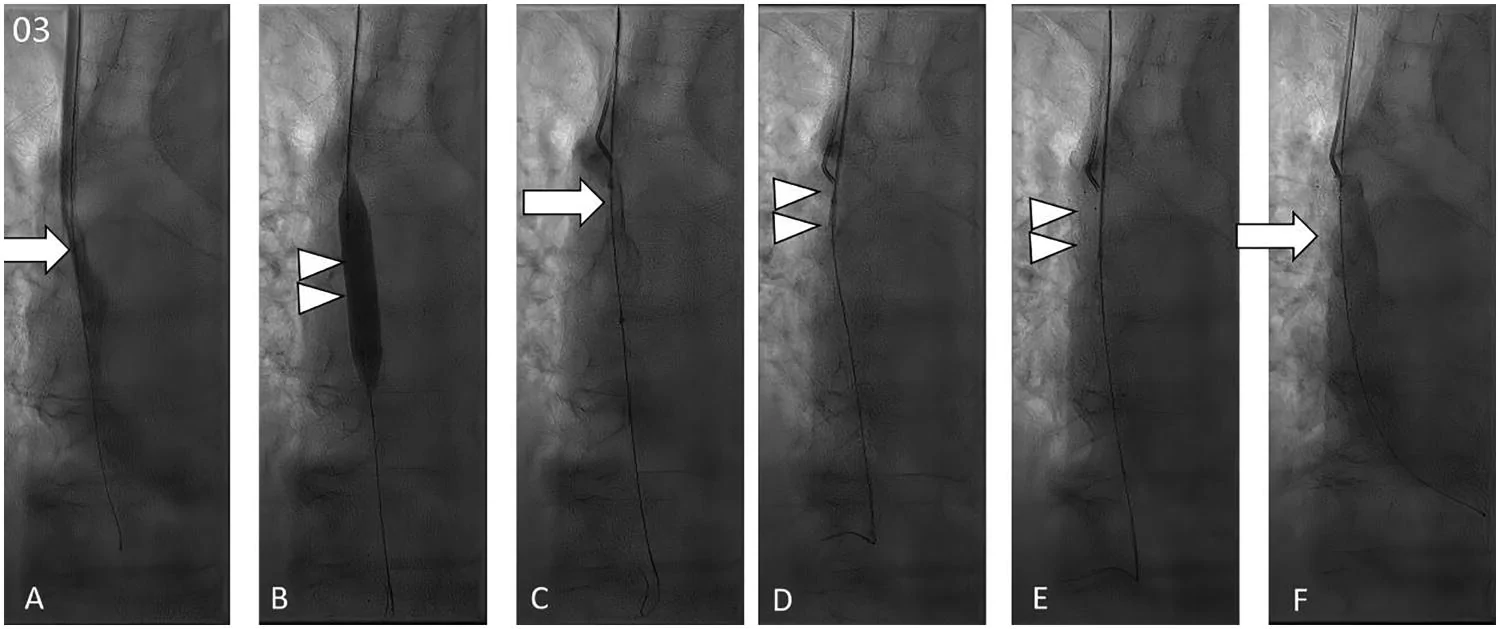
Stent placement at superior vena cava. A. Digital subtraction angiography showed superior vena cava stenosis (arrow). B. Balloon angioplasty is performed at the stenosis location (triangle). C. Venography after balloon angioplasty showed that the stenosis persists (arrow). D&E. A stent is placed at the stenosis location (arrow). Venography showed the superior vena cava flow recovered (arrow) after stent placement.

After catheter placement or exchange, all catheters were locked with standard heparin solution (1000 U/mL) after each hemodialysis session according to our institutional protocol.

### Follow-up and reintervention

After the procedure, patients received routine clinical monitoring according to a standardized protocol, which included: (1) monthly clinical assessment (including catheter outlet assessment and central venous stenosis-related symptoms (edema, varicose veins) assessment); (2) hemodialysis access flow monitoring according to Kidney Disease Outcomes Quality Initiative (KDOQI) guidelines to detect dysfunction; (3) Doppler ultrasound examination when catheter dysfunction was clinically suspected; and (4) digital subtraction angiography (DSA) for confirmed or persistent dysfunction. We recorded any reintervention due to catheter dysfunction and the time interval between procedures.

### Statistical analysis

We calculated left ventricular mass index (LVMI) using the following formulas: Left ventricular mass (LVM) = 0.8 * 1.04 * [(IVS + LVID + PWT)^3 – LVID^3] + 0.6. Body surface area (BSA) = 0.007184 * height^0.725 * weight^0.425. LVMI = LVM/BSA (IVS: interventricular septal thickness; LVID: left ventricular internal diameter; PWT: posterior wall thickness).

We analyzed the distribution of continuous variables. Data are presented as mean ± standard deviation or median (interquartile range), based on normality. Categorical variables are shown as percentages. We used Pearson or Spearman correlation tests to explore relationships between echocardiographic parameters and blood pressure, based on data normality. We used the paired sample T test or Wilcoxon rank-sum test to compare blood pressure before and after the intervention. We analyzed catheter survival using the Kaplan–Meier method and Cox regression analysis. Catheter survival was defined as the time from the index procedure to catheter removal or reintervention due to dysfunction, corresponding to primary patency. Patients were censored at the time of death, loss to follow-up, or study end. We performed all analyses using R Studio software (version 2024.09.0 + 375).

## Results

### Patient characteristics

A total of 123 patients with central vein occlusion were included in this study. There were 36 males and 87 females. The mean age was 61 years. Eleven patients (8.9%) initially commenced renal replacement therapy with peritoneal dialysis but later transitioned to hemodialysis due to peritoneal membrane failure. They later transitioned to hemodialysis due to peritoneal membrane failure. Five patients (4.0%) had a history of kidney transplant with subsequent graft failure. The mean duration of hemodialysis was 7.86 years. The mean duration of tunneled cuffed catheter use was 6.02 years. Thirty-four patients (27.6%) had a history of femoral vein catheterization, which in our center is generally used as the initial access for unscheduled or urgent hemodialysis initiation. Seventy-two patients (58.5%) had prior arteriovenous fistula or arteriovenous graft surgery. Fifty-one patients (41.4%) had symptoms related to central vein occlusion or stenosis. These symptoms included hypotension (*n* = 18, 14.6%), venous distension (*n* = 13, 10.5%), and edema (*n* = 19, 15.4%). Forty-two patients used antiplatelet medications. These included dipyridamole (*n* = 29, 23.6%), clopidogrel, and aspirin, used alone or in combination. Six patients used rivaroxaban for anticoagulation. Table 1 shows detailed patient characteristics.

**Table 1.** Baseline characteristics of patients.

Parameter/Indicator	Results
Age (years)	61.02 ± 13.66
Male	36 (29.2%)
Height (cm)	157 ± 0.08
Weight (kg)	55.29 ± 10.35
BMI (kg/m²)	22.42 ± 4.08
Central venous stenosis symptoms	51 (41.4%)
Hypotension	18 (14.6%)
Edema	19 (15.4%)
Varicose veins	13 (10.5%)
Prior peritoneal dialysis	11 (8.9%)
Prior kidney transplantation	5 (4.0%)
Prior femoral vein catheterization	34 (27.6%)
Prior arteriovenous fistula	72 (58.5%)
Hemodialysis vintage (years)	8 (4, 11)
Catheter usage duration (years)	5 (3, 8)
Comorbidities	
Heart failure, n(%)	14 (11.3%)
Diabetes mellitus, n(%)	24 (19.5%)
Coronary artery disease, n(%)	17 (13.8%)
Peripheral vascular disease, n(%)	7 (5.6%)
Stroke, n(%)	7 (5.6%)
Regular medication	
Antihypertensive drug^a^	57 (46.3%)
Calcium channel blockers (CCB)	38 (61.4%)
Beta-blockers	16 (28.1%)
ARB (angiotensin II receptor blockers)	7 (12.3%)
ARNI (sacubitril/valsartan)	6 (10.5%)
Alpha-blockers (terazosin)	6 (10.5%)
Active vitamin D	47 (38.2%)
Calcium supplements	7 (5.6%)
Cinacalcet	18 (14.6%)
Phosphate binders	59 (47.9%)
Antiplatelet agents	42 (34.1%)
Anticoagulants	6 (4.8%)

^a^ Percentages are based on the 57 patients using antihypertensive drugs. Each patient may be using one or more antihypertensive drugs, so the sum of percentages across drugs/drug classes may exceed100%.

### Central vein occlusion and stenosis

DSA diagnosed superior vena cava occlusion in 78 patients (63.4%), brachiocephalic vein occlusion in 92 patients (74.8%), and inferior vena cava occlusion in 28 patients (22.8%). In addition, 49 patients (39.8%) had angiographic evidence of venous stenosis without complete occlusion in at least one central vein. In this study, “occlusion” refers to complete luminal obstruction, whereas “stenosis” refers to partial luminal narrowing without complete blockage; these terms are not used interchangeably. Regarding laterality, lesions (including both stenosis and occlusion) were more frequently observed on the right side in 86 patients (69.9%), whereas 13 patients (10.6%) had left-sided lesions; the remaining patients had bilateral involvement or DSA did not report the specific left or right location of the affected vessel. Right atrial thrombus was found in 22 patients (17.8%). Fibrin sheath around the catheter tip was identified in 47 patients (38.2%). Azygos vein compensation was present in 18 patients (14.6%). Besides, catheter-related bloodstream infections, defined by positive catheter tip or blood culture, occurred in 8 patients (6.5%). No complications such as hematoma, pneumothorax, bleeding or venous perforation occurred during the operation.

### Catheter exchange and endovascular treatment

Ninety-seven patients (78.9%) underwent catheter exchange at the original site. Twenty-six patients (21.1%) received a new catheter at a different site. Fifteen patients underwent superior vena cava puncture. Among these 15 patients, the occluded sites included the brachiocephalic vein in 12 patients, the superior vena cava in 12 patients, and the inferior vena cava in 10 patients (these numbers overlap because most of these 15 patients had concurrent occlusions at multiple sites rather than a single isolated lesion). Balloon angioplasty was performed in the superior vena cava (*n* = 64), brachiocephalic vein (*n* = 77), and inferior vena cava (*n* = 28). Twelve patients received a stent.

### Follow-up

All 123 patients had available post-procedure hemodialysis data. The median follow-up and catheter survival time was 735 days (range 3–2828 days). During follow-up, 48 patients (39.0%) experienced recurrent catheter dysfunction requiring reintervention. The median time to reintervention was 255 days (range 3–1768 days). Central vein occlusion was confirmed in all patients during the second procedure. Table 2 shows these results.

**Table 2.** Procedure details and outcomes.

Parameter	N (%)
Angiographic lesions	
Atrial thrombus	22 (17.8%)
Fibrin sheath	47 (38.2%)
Azygos vein compensation	18 (14.6%)
Thrombus (including atrial)	30 (24.3%)
Interventional procedures	
Catheter exchange	97 (78.8%)
New catheter insertion (different site)	26 (21.1%)
Superior vena cava puncture	15 (12.2%)
Balloon dilatation performed	102 (82.9%)
Stent placement	12 (9.7%)
Long-term follow-up	
Reintervention	48 (39.0%)
Time to reintervention (days)	255 (65, 708)
≥2 reinterventions after this procedure	18 (14.6%)

Due to the severely imbalanced sample sizes between the comparison groups for balloon angioplasty (102 vs. 21) and stent placement (12 vs. 111), prognostic analyses for these two interventions were not performed. For other indicators, Kaplan–Meier survival analysis showed that the presence of atrial thrombus and fibrin sheath were significantly associated with shorter catheter survival time. The Kaplan–Meier survival curves are shown in Figure 4. Univariate Cox regression analysis also showed a significant association between atrial thrombus, fibrin sheath, and shorter catheter survival. Multivariate Cox regression analysis confirmed this significant association. We found no significant association between catheter survival and other factors, including sex, age, BMI, hemodialysis vintage, catheter use duration, hypertension, diabetes mellitus, use of active vitamin D, phosphate binders, antiplatelet agents (dipyridamole, clopidogrel, aspirin), and rivaroxaban.

**Figure 4.**
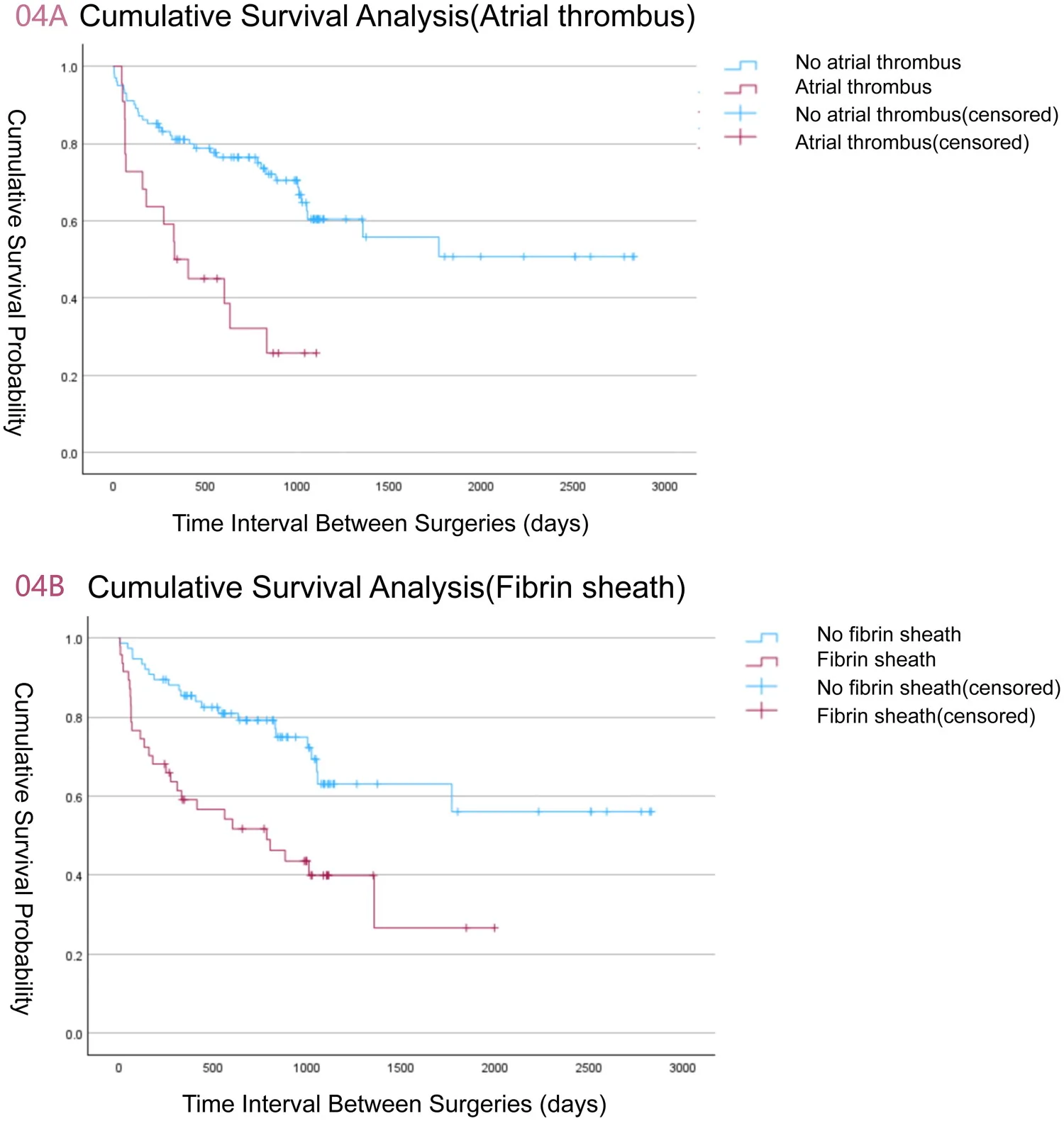
Kaplan–Meier survival curves. A. Kaplan–Meier survival curves for catheter survival in patients with and without atrial thrombus. B. Kaplan–Meier survival curves for catheter survival in patients with and without fibrin sheath.

### Echocardiographic parameters

Fifty-one patients had echocardiography data before the endovascular procedure (According to the test, there was only significant difference in gender between the 51 patients and the remaining 72 patients (*p* = 0.048 male 19.6% vs 36.1%), and there was no significant difference in other baseline indicators). Right ventricular basal diameter was reduced (<24 mm) in 50 patients (98.0%). Left ventricular mass index (LVMI) was increased in 11 patients (21.6% of the 51 patients). Based on the echocardiographic reports, ventricular hypertrophy was present in 27 patients (52.9% of the 51 patients). Atrial enlargement was present in 28 patients (54.9% of the 51 patients). Left ventricular ejection fraction (EF) was reduced (<60%) in 8 patients (15.7% of the 51 patients) and increased in 15 patients (>70%) (29.4% of the 51 patients). The remaining patients had normal EF. Detailed data are shown in Supplementary Table 1.

### Blood pressure

Seventy-eight patients (63.4%) had a history of hypertension. Among them, 21 patients had normal blood pressure on admission without antihypertensive medications. Fifty-seven patients were still taking one or more antihypertensive drugs. Detailed medication information can be seen in Table 1. Hypotension was observed in 18 patients (14.6%) on admission. Although CVO may contribute to this condition, hypotension in this population is often multifactorial and not uniformly attributable to central venous occlusion alone. Among these hypotensive individuals, half experienced blood pressure elevation after endovascular intervention. Nevertheless, no statistically significant overall between-group difference was detected in blood pressure before versus after procedures. Detailed blood pressure data are shown in Supplementary Table 2.

## Discussion

We included 123 hemodialysis patients with tunneled cuffed catheter dysfunction and central venous occlusion. We reported our findings on treatment methods, patient characteristics, and long-term prognosis of these patients.

Although stenosis is a well-recognized precursor to thrombosis in this population, our analysis focused primarily on complete occlusions, as these represented the lesions that precluded standard catheter placement and dictated the need for salvage intervention. Nevertheless, we observed concurrent stenosis in 49 patients (39.8%), highlighting the frequent coexistence of these two entities.

It is difficult to establish dialysis access in patients with central venous occlusion. Conventional salvage approaches for CVO, including HeRO graft, translumbar/transhepatic catheter and sharp recanalization, are limited by unsatisfactory patency or high procedural complications. For example, HeRO presented with comparatively low 1-year primary and secondary patency rates (28.7%-58.2%, 64.5%-90.6%, respectively) [14]. Besides, Harter et al. have performed 73 cases of HeRO graft implantation in their center since 2013, and they found that after an implantation a reintervention for acute thrombosis is necessary 2–3 times per year [15]. Translumbar catheter showed a varied 1-year patency of 17% to 88.7% [16–18]. And transhepatic catheter showed a relatively short initial device service interval (27–290 days) [17]. For sharp recanalization, due to the sharp nature of the surgical instruments, operation-related complications are more frequent. Life-threatening complications, such as hemothorax and acute cardiac tamponade, are also more likely to occur [13]. In summary, the currently available salvage options are constrained by various limitations. Peritoneal dialysis (PD) was considered as an alternative modality for patients with exhausted vascular access, provided no contraindications were present. However, only a minority of eligible patients ultimately opted for PD, mainly due to patient preference for in-center hemodialysis, concerns regarding the burden of home-based self-care, or poor general condition precluding PD eligibility. In our cohort, only 11 patients (8.9%) had a history of PD, all of whom eventually transitioned to hemodialysis due to peritoneal membrane failure. Kidney transplantation, when feasible, remains the optimal definitive treatment for eligible patients with exhausted vascular access, though it is limited by organ availability and waitlist time [19,20]. In contrast, our regimen prioritized routine internal jugular catheter revision, with direct superior vena cava (SVC) puncture reserved for patients unsuitable for standard access; 15 such complex CVO patients received SVC puncture. Our cohort achieved a favorable median catheter survival of 735 days, with only 39.0% requiring reintervention after a median of 255 days and no immediate perioperative complications. Collectively, our protocol offers a safe and reliable alternative for patients lacking feasible routine access routes.

For patient characteristics, in our study, the number of female patients with central venous occlusion was more than twice that of male patients, which may suggest central venous occlusion be more common in female hemodialysis patients. The higher incidence of thrombosis in women may be the reason accounting for it [21]. However, in some small studies, the proportion of women is only slightly more than men [4,7,22]. And a previous study involving 117 hemodialysis patients with SVC stenosis also reported an equal sex ratio (female:male 58:59) [23]. Whether sex differences exist in hemodialysis patients with central venous occlusion needs further study. We also reported that 41.4% patients had central venous occlusion and stenosis-related symptoms. There is no large-scale study to count the proportion of symptomatic patients. In some studies that included only a dozen patients with central venous occlusion, the proportion of symptomatic patients reported was small [24]. Besides, our study found that the proportion of patients with brachiocephalic vein occlusion was the highest among patients with central venous occlusion (74.8%). Pasqui et al. reported similar results (70.4%) [25]. In the study of Cildag, the proportion of brachiocephalic vein occlusion was also the largest (59.1%) [4]. These findings seem to indicate that dialysis patients are most prone to brachiocephalic vein occlusion.

In the patients with catheter dysfunction included in this study, right atrial thrombus was found in 22 patients (17.8%). Previous research reported the incidence rate of catheter-related right atrial thrombus (CRAT) varies from 2 to 29% in patients with TCC [26]. Fibrin sheath was identified in 47 patients (38.2%) in our study. Previous studies have reported there were 6% of patients with long-term indwelling dialysis catheter having fibrin sheath [27]. However, in our study, fibrin sheath was diagnosed by DSA, while the fibrin sheath in this previous study was mainly diagnosed by computed tomography, and there may be some patients who also have fibrin sheath but remain undetected. We found that the presence of atrial thrombus and fibrin sheath was significantly associated with shorter catheter survival, which was confirmed by both Kaplan–Meier and Cox regression analyses. It has been reported in previous studies that thrombosis or fibrin sheath formation was the complication most often limiting catheter lifespan, especially beyond the first week after insertion [28,29]. Previous research reported catheter-related thrombosis can lead to the loss of vascular access in the relevant vein in up to 30%-40% of patients. Besides, a study by Sehgal et al. showed that catheter-related atrial thrombus was significantly (*p* < 0.001) and independently related to inadequate dialysis dose delivery [30]. A systematic review of 8 studies reported that interventions such as thrombolysis, fibrin sheath disruption, and catheter exchange are all effective for immediately restoring patency. There is no evidence to support physical intervention over the use of pharmaceutical agents in the acute setting. Long-term catheter survival may be improved by fibrin sheath disruption and is probably superior following catheter exchange [31]. However, one study indicated anticoagulation has no significant benefit in delaying sheath formation in patients with TCC. Therefore, the fibrin sheath disruption is preferred for fibrin sheath [32]. Patients in our study were only treated with catheter replacement or re-catheterization, and the results showed that it was also effective. The above results showed that thrombosis and fibrin sheath are common complications of TCC implantation, and effective methods should be adopted as soon as possible.

However, our study indicated other factors, such as age, sex, diabetes, anticoagulant and antiplatelet drug use were not significantly associated with catheter survival. It had been reported by a study that female sex (HR = 1.497, 95% CI: 1.119–2.002; *p* = 0.007) was associated with an increased rate of catheter dysfunction [33]. In terms of gender, our study did initially include significantly more women than men with catheter dysfunction, which is consistent with this result. Previous studies have also consistently demonstrated that female sex is an independent risk factor for thrombosis in hemodialysis patients. A recent meta-analysis of 17 studies showed that female sex was associated with a 48.1% increased risk of vascular access thrombosis (OR = 1.481, 95% CI: 1.177–1.862, *p* < 0.001) [21]. Mohazzab et al. found that female sex was associated with a subdistribution hazard ratio of 1.81 for catheter thrombosis (*p* < 0.05) in a competing risk analysis of 466 patients [34]. Furthermore, VIVALDI study of 626 hemodialysis patients identified female sex as an independent predictor of venous thromboembolism (OR = 1.90, 95% CI: 1.07–3.36, *p* = 0.029) [35]. The higher risk of vascular access thrombosis in women may be the reason why women have shorter catheter survival time. And perhaps because the proportion of thrombus in patients after endovascular treatment is reduced, the gender difference is no longer significant, which still need more research to confirm.

For age, one study showed every 10-year increment in age (HR = 1.243, 95% CI: 1.123–1.376; *p* < 0.001) were associated with an increased rate of catheter dysfunction [36]. However, in another study of 472 incident hemodialysis patients, Shingarev et al. found that catheter patency was not associated with patient age [37]. Although the existing evidence remains controversial, from the perspective of clinical decision-making, increasing age is indeed an important consideration, as older TCC users are at a higher risk of death than younger users and have fewer opportunities to transition to a fistula. Another previous study including 361 patients with TCC indicated diabetes mellitus was an important predictor of catheter dysfunction and was significantly correlated with catheter extraction [37]. The small number of patients with diabetes (only 19 cases) in our study may be the reason for our negative results. Diabetes may reduce the catheter survival rate by increasing the risk of infection and thrombosis. Finally, within the limitations of our retrospective data (particularly the lack of dosage, adherence, and laboratory target information), we did not detect a significant association between chronic anticoagulation or antiplatelet therapy and catheter survival. This is consistent with a previous study reporting that chronic anticoagulation and antiplatelet treatment did not affect the rate of TCC dysfunction or replacement [38]. However, given the aforementioned data limitations, this finding should be interpreted cautiously and does not exclude a potential benefit of these therapies when used at appropriate doses and with adequate monitoring.

Echocardiographic data showed that most patients had reduced right ventricular basal diameter (98.0%). Left ventricular mass index was increased in 40.9% of patients with available data. Previous studies have demonstrated that the cardiac changes associated with central venous occlusion in hemodialysis patients are distinct from typical heart failure patterns [39,40]. Horita et al. evaluated cardiac function before, immediately after, and on the day following PTA for central venous lesions in six hemodialysis patients. They found that right atrial and right ventricular diameters increased significantly immediately after PTA, reflecting acute volume overload from restored venous return, but these changes were transient and completely recovered by the following day [38]. Horita’s findings support our explanation that the reduction of the right heart is caused by obstruction. The right ventricle tends to decrease in size due to restricted filling, as elevated central venous pressure from the occlusion creates a functional barrier to venous return, while the negative pressure from dialysis aspiration further limits right ventricle preload. Conversely, the left ventricle often enlarges as a compensatory response to chronic volume overload, driven by neurohormonal activation and interdialytic fluid retention, despite reduced pulmonary venous return [40]. Additionally, a leftward shift of the interventricular septum (D-sign) impairs left ventricular compliance, promoting concentric hypertrophy and dilation [40]. This paradoxical pattern (right ventricular underfilling with left ventricular volume overload) necessitates venous intervention rather than aggressive ultrafiltration.

There are 63.4% of patients included in our study had a history of hypertension, which is lower than the proportion of patients with hypertension in general hemodialysis patients (74.6%-90%) or hemodialysis patients with central venous stenosis (78.8%) [41–43]. Our study found that there was no significant difference in overall blood pressure before and after endovascular treatment in the included patients. No previous studies had clearly pointed out that endovascular treatment and replacement of catheters can improve blood pressure in hemodialysis patients with CVO, they only showed that endovascular treatment can improve cardiac output in such patients. However, our study did not find a correlation between cardiac output and pressure in such patients. 18 of the patients we included had hypotension on admission. Of note, while CVO may contribute to hypotension by reducing venous return and cardiac preload, this condition is often multifactorial. Other factors such as autonomic dysfunction, excessive ultrafiltration, and cardiac impairment may also be involved. Only 9 of these 18 patients showed blood pressure improvement after endovascular intervention, suggesting that CVO was not the sole cause. The poor blood pressure response in the remaining patients may be due to irreversible damage from prolonged central venous occlusion.

This study has some limitations. First, this was a single-center retrospective study with all endovascular procedures performed by a single interventionalist. While this ensures procedural consistency, it also limits the generalizability of our findings to other clinical settings or to operators with different levels of experience and technical approaches. The outcomes observed in our cohort may not be directly reproducible in centers with different patient populations, practice patterns, or institutional resources. Therefore, future multicenter prospective studies with larger sample sizes and multiple operators are warranted to validate our findings and to better define the optimal treatment strategy for this complex patient population. Second, for both balloon angioplasty (102 vs. 21) and stent placement (12 vs. 111), the comparison groups were severely imbalanced. We did not perform prognostic analyses for these interventions, and no conclusions regarding their impact on catheter survival can be drawn from this dataset. Third, for the anticoagulation of such patients, the use of antiplatelet and anticoagulant agents was heterogeneous (multiple drugs combined), and we did not collect data on dosage, adherence, or laboratory targets such as INR, precluding adequate adjustment for their potential impact on thrombosis and catheter survival. This limits our ability to draw definitive conclusions regarding the true effect of these medications on catheter outcomes. Additionally, our intraprocedural use of unfractionated heparin in patients with thrombosis was based on clinical experience rather than standardized guidelines, as KDOQI does not provide specific recommendations for prophylactic anticoagulation during guidewire manipulation across occluded central veins. Finally, we acknowledge that echocardiographic data were available for only 51 of the 123 patients (41.5%). Comparison of baseline characteristics between this subgroup and the remaining 72 patients revealed no significant differences except for a lower proportion of males (19.6% vs. 36.1%, *p* = 0.048), suggesting that selection bias cannot be entirely excluded. Therefore, our echocardiographic findings should be interpreted with caution and considered hypothesis-generating. Prospective studies with systematic echocardiographic assessments are warranted to confirm these cardiac remodeling patterns.

In conclusion, our treatments for CVO in hemodialysis patients with TCC are safe and effective. Thrombosis and fibrin sheath are common complications of TCC implantation, and effective methods should be adopted as soon as possible. Chronic anticoagulation and antiplatelet treatment may not affect the rate of TCC prognosis. Cardiac evaluation revealed a unique pattern of right ventricular underfilling with left ventricular volume overload, supporting venous intervention over aggressive ultrafiltration. These findings highlight the need for early intervention and long-term management strategies beyond procedural success.

Sichuan Science and Technology Program, 2024NSFSC1504**; **National Natural Science Foundation of China, 82200837.

## Supplementary Material

Clean Supplementary Table.docx

## Data Availability

The original data of this study were stored in an online database. Mendeley Data, doi: 10.17632/7f9vf63ynz.1
