# Supplementary material for: Treatment, characteristics and prognosis of central venous occlusion in hemodialysis patients with tunneled cuffed catheter dysfunction
Source: Ren Fail. 2026 Sep 27;48(1):2732516. doi: 10.1080/0886022X.2026.2732516 (PMC13629761; doi:10.1080/0886022X.2026.2732516)
Supplement: Supplementary file 1 [file IRNF_A_2732516_SM2458.docx]

**Supplemental Table 1. Echocardiographic Findings (n=51 10 males and 41 females)**

| Parameter / Indicator | Results |
| --- | --- |
| Right ventricular basal diameter (mm) | 20 ± 2.06 |
| Left ventricular end-diastolic volume index (ml/m²) | Female: 98.50 (82.00, 108.75) Male: 113.50 (102.50, 124.00) |
| Left ventricular end-systolic volume index (ml/m²) | Female: 32.50 (25.00, 38.75) Male: 38.50 (32.50, 50.75) |
| Stroke volume (ml) | Female: 62.55 ± 11.61 Male: 75.40 ± 12.73 |
| Left ventricular mass index (LVMI, g/m²) | Female: 85.25 (77.43, 96.20) Male: 92.10 (90.65, 104.25) |
| Left ventricular ejection fraction (%) | 66.00 (60.75, 71.00) |

**Supplemental Table 2. Blood Pressure Data (n=123)**

| Parameter / Indicator | Results |
| --- | --- |
| Concomitant hypotension | 18 (14.6%) |
| Concomitant hypertension | 78 (63.4%) |
| Preoperative systolic blood pressure (mmHg) | 130.42 ± 26.48 |
| Preoperative diastolic blood pressure (mmHg) | 79.72 ± 13.98 |
| Preoperative heart rate (bpm) | 85.00 (75.00, 95.50) |
| Postoperative day 1 systolic blood pressure (mmHg) | 128.66 ± 24.09 |
| Postoperative day 1 diastolic blood pressure (mmHg) | 78 (70.50, 86.00) |
| Use of antihypertensive drugs | 57 (46.3%) |
